# Gut Bacterial Composition and Functional Potential of Tibetan Pigs Under Semi-Grazing

**DOI:** 10.3389/fmicb.2022.850687

**Published:** 2022-04-07

**Authors:** Hui Niu, Xi-Ze Feng, Chun-Wei Shi, Di Zhang, Hong-Liang Chen, Hai-Bin Huang, Yan-Long Jiang, Jian-Zhong Wang, Xin Cao, Nan Wang, Yan Zeng, Gui-Lian Yang, Wen-Tao Yang, Chun-Feng Wang

**Affiliations:** Jilin Provincial Key Laboratory of Animal Microecology and Healthy Breeding, Key Laboratory of Animal Production and Product Quality Safety of the Ministry of Education, Jilin Provincial Engineering Research Center of Animal Probiotics, College of Veterinary Medicine, College of Animal Science and Technology, Jilin Agricultural University, Changchun, China

**Keywords:** gut bacteria, Tibetan pigs, bacterial functions, growth stages, genders

## Abstract

Gut bacterial community plays a key role in maintaining host health. The Tibetan pig (*Sus scrofa*), an ancient breed in China, has been known for its high adaptability to harsh environments and for its meat quality. To understand the underlying mechanisms facilitating to shape these unique features, in this study, *16S rRNA* sequencing using pigs feces and subsequent bacterial functional prediction were performed. Also, the gut bacteria of two other breeds of pigs, Barkshire and Landrace, were examined for comparison. It was revealed that the structure of bacterial community in Tibetan pigs appeared to be more complex; the relative abundances of dominant bacterial families varied inversely with those of the other pigs, and the proportion of *Firmicutes* in Tibetan pigs was lower, but *Bacteroides, Fibrobacterota*, *Lachnospiraceae*, *Oscillospiraceae*, and *Ruminococcaceae* were higher. Bacterial functional prediction revealed that the dominant flora in the Tibetan pigs was more correlated with functions regulating the hosts’ immune and inflammatory responses, such as NOD-like_receptor_signaling_pathway and vitamin metabolism. In addition, in Tibetan pigs, the taxonomic relationships in the gut bacteria on day 350 were closer than those on earlier stages. Furthermore, gender played a role in the composition and function of bacterial inhabitants in the gut; for boars, they were more correlated to drug resistance and xenobiotics metabolism of the host compared to the sows. In sum, our preliminary study on the gut bacterial composition of the Tibetan pigs provided an insight into the underlying host–microorganism interactions, emphasizing the role of intestinal bacteria in the context of modulating the host’s immune system and host development.

## Introduction

Gut microflora plays a vital role in maintaining the health of hosts (Ross et al., 2019). Previous studies revealed that it not only was implicated in the promotion of hosts’ mucosal and systemic immunity and hematopoiesis (Staffas et al., 2018), but also was associated with modulation of the host’s hypertension-linked metabolic disorders and inflammation (Li J. et al., 2021). In addition, gut microbiome was recognized as an acquired bacterial defense system (Ross et al., 2019). The interactions between gut microbiota and host immunity exerted effective suppressions of helminth-evoked colitis (Shute et al., 2021). Recent research suggested that adjustment of intestinal microbiota could improve efficacies of treatments of colorectal cancer (Si et al., 2021). In contrast, dysregulated intestinal bacteria and fungi could lead to the loss of intestinal barrier and therefore enhanced disease susceptibility in hosts (Chopyk and Grakoui, 2020). It was reported that alleviation of the imbalance of intestinal microbiota by colonic dialysis could protect the renal function in patients with pre-dialysis chronic kidney disease (Li Y. et al., 2021).

Compared with rodents, swine was considered as a more ideal model for studying human diseases due to its narrow disparities in organ characteristics compared to those of humans (Gehrig et al., 2019). Porcine models were especially adopted for studies of lung cystic fibrosis in humans (Lu and Kolls, 2021) and were also used in the field of pathogenesis of neurodegenerative (Yan et al., 2018) and cardiovascular (Hinkel et al., 2015; Fan et al., 2020; Zhao et al., 2021) diseases. Furthermore, pigs’ omnivorous eating behavior contributed to its role in the study of gut microbiota. It was demonstrated that intestinal *Prevotella copri* was a factor for host obesity and fat accumulation (Chen C. et al., 2021). Both biomedical research and agriculture would benefit from enhanced understanding of the pig gut microbiome (Wylensek et al., 2020).

The Tibetan pig, a prototypic breed of pig in China with features of adapting to a wide range of breeding conditions, especially the harsh plateau environment (Ma et al., 2019), was known to possess a high lean meat ratio, and its meat was recognized as green food owing to free range, which made it particularly popular in the high-end market (Huang et al., 2021). As miniature pigs, Tibetan pigs are similar in size to humans, which makes them a superior model for human studies (Lunney, 2007). Previous studies (Cheng et al., 2015) compared respective immune performances in Tibetan and Yorkshire pigs, showing that a stronger innate immunity was exerted in Tibetan pigs, which could trigger comparatively decent local or system immune responses in the hosts to protect against invading pathogens. Interestingly, when Tibetan pigs were introduced and bred in other areas, none ended up with non-acclimatization or sickness (Wu et al., 2007). A DNA recombinant vaccine developed with Tibetan pig *IL-23* gene was shown to enhance the overall immune responses of pigs against infections caused by Porcine circovirus type 2 (Xiao et al., 2020).

There have been some studies on the intestinal microbes of Tibetan pigs. It is reported that there are differences in the intestinal microbes of Tibetan pigs in different living environments and growth stages (Jiang et al., 2018; Zeng et al., 2020). The rich carbohydrate-degrading enzymes in the gut microbes of Tibetan pigs can bring us more benefits in the future microbial production of industrial enzymes (Zhou et al., 2020). To date, limited knowledge was available on the gut bacteria of Tibetan pigs. In this study, *16S rRNA* metagenomic analysis of fresh pigs feces was performed to explore the relation between gut microbiome and host health (Martinez-Guryn et al., 2019). In addition, comparisons of the gut bacteria between Tibetan and Berkshire pigs, which were raised under semi-grazing conditions, and Landrace pigs raised in large-scale houses were conducted to understand the functions of the gut bacterial flora in Tibetan pigs. Besides, gut bacteria underwent dynamic changes in the process of growth (Bäckhed et al., 2015), and were distinct in structure, composition, and diversity between male and female hosts (Wang et al., 2020), which were also investigated in this study to gain an insight into functional gut bacteria in Tibetan pigs.

## Materials and Methods

### Animals and Fecal Sample Collection

In this study, the Tibetan pigs (*Sus scrofa*) used were a cross between hybrid sows (*S. scrofa*) and purebred boar (*S. scrofa*); the parental sows were a progeny of Tibetan pigs crossed with other black pig breeds, including Beijing black pigs and hybrid Tibetan pigs. These second-generation of Tibetan pigs employed in this study could be degenerated into wild type, accelerating farrowing rates, which was in line with actual production needs. Currently, most Tibetan pigs circulating in the market are this second-generation Tibetan pig.

Forty-eight fresh fecal samples were collected from a Tibetan pig farm in Shanxi Province, China (37°30′N, 113°83′E). In this study, three levels of comparisons were performed, including pigs of different breeds, Tibetan pigs of different growth stages, and Tibetan pigs of different sexes. Fresh feces of Landrace pigs were collected under housing conditions, while those of Berkshire pigs and Tibetan pigs were collected under semi-grazing conditions. They are fed all-natural cornmeal and wheat bran. In addition, Berkshire pigs and Tibetan pigs can eat freely on the mountain. For the comparison of gut bacteria between breeds, pigs of different breeds were raised to about 350 days of age, and their feces were collected. The growth of pigs are usually differentiated into phases of lactation, weaning, and fattening. Naturally, the lactation and growing time of Tibetan pigs are relatively long; accordingly, three time points, days 7, 60, and 350, were selected for evaluating the gut bacterial composition in Tibetan pigs. In addition, the feces of Tibetan boars, pregnant sows, and suckling sows were collected and detected. Six parallel samples in each group were set up to eliminate individual differences. Separate stool samples were collected in individual sterilized containers after rectal stimulation and then immediately transported to laboratory on dry ice, and stored at −80°C.

### DNA Extraction

The fecal samples were subjected to DNA isolation with PowerSoil DNA Isolation Kit (MoBio Laboratories, Carlsbad, CA) following the manufacturer’s instruction. Purity and quality of the genomic DNA were checked by a NanoDrop spectrophotometer (Thermo Fisher Scientific, Massachusetts, United States).

### Polymerase Chain Reaction Amplification

After quantification with NanoDrop measurement, 30 ng of DNA was used for Polymerase Chain Reaction (PCR) amplification. The variable region of *16S rDNA* V3–V4 (338–806) was used to design primers as follows: 338F (5′-ACTCCTACGGGAGGCAGCAG-3′) and 806R (5′-GGACTA CNNGGGTATCTAAT-3′) (Munyaka et al., 2015). PCR was performed in triplicate in 25-μl volumes containing 2.5 μl of 10 × Pyrobest Buffer, 2 μl of 2.5 mM dNTPs, 1 μl of each primer (10 μM), 0.4 U of Pyrobest DNA Polymerase (TaKaRa, Dalian, China), and 15 ng of template DNA (Chen H. et al., 2021). PCR conditions were pre-set as: pre-denaturation was performed at 94°C for 3 min, followed by 25 cycles of denaturation at 94°C for 30 s, then annealed at 50°C for 30 s and extended at 72°C for 60 s (Xing et al., 2021). After this, agarose gel electrophoresis was performed to detect the specificity of the amplification results. The gel was prepared at a concentration of 1%, running at a voltage of 170 V, for 30 min.

### Sequencing Library Preparation and Sequencing

Sequencing reactions were carried out at Allwegene Company in Beijing, China using Illumina Miseq PE300 platform (Illumina, Inc., CA, United States).

The off-machine data were divided into samples according to the barcode sequence through the QIIME (v1.8.0), and Pear (v 0.9.6) was used to filter and splice the data (Visscher et al., 2019). Ambiguous bases and primer mismatches were removed. The minimum overlap was set as 10 bp, and mismatch rate was set as 0.1. Then, Vsearch (v 2.7.1) was employed to remove sequences less than 230 bp in length, and the uchime method was used to compare and remove chimera sequences according to Gold Database. After these, Uparse algorithm of Vsearch (v 2.7.1) was used for picking up operational taxonomic units (OTUs) cluster, with 97% similarity as the cutoff (Edgar, 2013). Ribosomal Database Project (RDP) Classifier tool was used to classify all sequences into different taxonomic groups against the SILVA128 database (Cole et al., 2009); 70% was used as the confidence threshold.

### Diversity and Structural Analysis of the Bacterial of Fecal Samples

QIIME1 (v1.8.0) was used to generate rarefaction curves, α indices (including Shannon, Simpson, Chao1 indices, goods_coverage, observed_species, and PD_whole_tree), and β diversity. The VennDiagram package of R (v3.6.0) was used for making Venn diagrams (Fouts et al., 2012), which allowed for visualizing the number of OTU groups of environmental samples and the overlap between samples or groups. GraphPad Prism (v 9.0.0) was used to estimate alpha-diversity and analyze the dominant phyla. To examine the similarity among Landrace, Berkshire, and Tibetan pigs, R (v3.6.0) was used to perform principal coordinate analysis (PCoA) based on Weighted Unifrace distance (Wang et al., 2012). Based on the species annotation and relative abundance results, R (v3.6.0) software was used to visualize the relative abundance of pig breeds and species composition as boxplots. Besides, the growth dynamics of Tibetan pigs and the composition of intestinal flora were visualized using R. In addition, R software was also used to analyze the histogram of the species composition of Tibetan pigs of different genders. Linear discriminant analysis (LDA) effect size (LEfSe) in galaxy was used to understand the bacterial differences between the breeds of pigs by coupling statistical analysis with examinations of biomarkers and their effect relevance. In this study, an LDA score of 4 was set as the threshold. In addition, to determine the taxonomic relations between bacterial communities in the gut bacteria, the relationship between OTUs was analyzed by Spearman correlation coefficient and R (v3.6.0) software, and the co-occurrence analysis was performed with Cytoscape (Wen et al., 2016). Functional-gene profile was inferred with software package Phylogenetic Investigation of Communities by Reconstruction of Unobserved States (PICRUSt) based on *16S rRNA* gene cluster survey along the samples accompanied by its relative abundance in each of the samples. R was used for heatmap visualization. The top 15 correlated pathways in Tibetan pigs at different growth stages were presented in this study. To determine the correlation between functional pathways and gender, the comparison was visualized with a histogram.

### Statistical Analyses

Metastats analysis was performed to determine the significant differences between groups. Kruskal–Wallis tests were conducted to calculate the differences between the means of independent data in multiple groups, and Wilcoxon tests were carried out to compare the differences between two paired groups. Besides, *t*-test and one-way ANOVA are also performed to calculate the significance of data.

## Results

### *16S rRNA* Sequencing Data

The *16S rRNA* sequencing generated 4,297,102 raw reads in total. Good_coverage index of the 48 samples in this study was greater than 99%. As shown in the curves of Rarefaction (Supplementary Figure 1A), Shannon-Wiener (Supplementary Figure 1B), Rank-Abundance (Supplementary Figure 1C), and species accumulation (Supplementary Figure 1D), the total initial sample reads increased with the sample sizes and then developed into stagnant stages, which indicated that the bacterial complexity in the communities reached its peak and would not enhance sample sizes, suggesting that both sample sizes and sequence reads were sufficient to cover the bacterial inhabitants in the communities.

### The Dominant Gut Bacteria in Tibetan Pigs and Its Potential Functions

**FIGURE 1:**
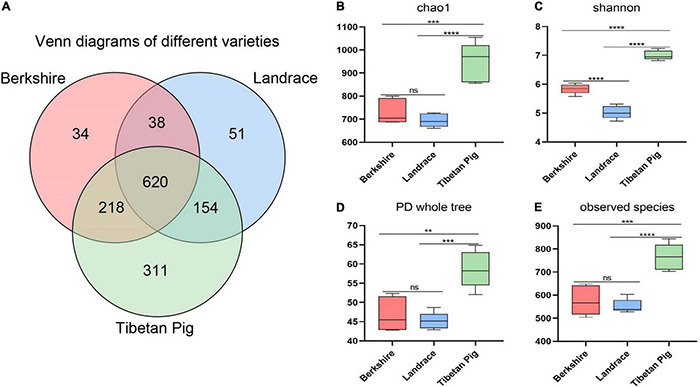
Venn map and alpha-diversity analysis. Venn map of Berkshire pigs, Landrace pigs, and Tibetan pigs on day 350 **(A)**. Chao1 observed number of species **(B)**. Shannon–Wiener index **(C)**. PD whole tree **(D)**. Observed species **(E)**. One-way ANOVA was employed for the statistical analysis (ns, non significant; **p < 0.01; ***p < 0.001; ****p < 0.0001).

Overall, 1,426 OTUs were identified in the fecal samples of Tibetan pigs by *16S rRNA* sequencing, of which 311 were unique. The number of OTUs in Tibetan pigs is significantly higher than those in Landrace and Berkshire pigs at age about 1 year (350 days) (Figure 1A). Landrace and Berkshire pigs produced 863 and 910 OTUs, respectively. Of the three breeds, the gut bacteria of Landrace and Berkshire pigs were shown to have a similar number of unique OTUs, 51 and 34, respectively. The estimations of alpha diversity of the bacteria for Tibetan pigs were shown to be significantly greater than those for Landrace and Berkshire pigs indicated by indices of Chao1, Shannon, PD_whole_tree, and observed OTUs (observed species) (Figures 1B–E), which suggested that the bacterial diversity of Tibetan pigs’ gut bacteria was significantly higher than those of the other two breeds, and the Landrace pigs’ was the lowest.

**FIGURE 2:**
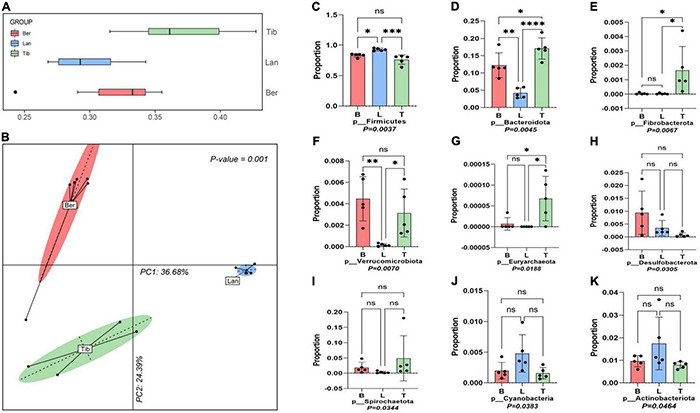
Boxplot, principal coordinate analysis (PCoA), and dominant phylum analysis. Boxplot **(A)** and Principal coordinate analysis (PCoA) based on weighted UniFrac Distances per pig fecal sample **(B)**. Dominant bacteria phyla of three different breeds of pigs were screened out by the Kruskal–Wallis test **(C–K)**. ns was considered insignificant; *p < 0.05 was considered as significant difference; ^**^p < 0.01, ^***^p < 0.001 and ^****^p < 0.0001 was taken as very significant difference.

Boxplot and PCoA based on the unweighted UniFrac distance of MetaOTUs showed clear differences between the community structures of the three breeds of pigs, and the Landrace pigs samples are most similar to each other (Figures 2A,B). The analysis showed that the samples of Tibetan pigs are more similar to the samples of Barkshire pigs than Landrace pigs (Figures 2A,B).

Nine phyla were screened out of the three gut bacterial communities after the flora data were subjected to Kruskal–Wallis tests (Figures 2C–K). *Firmicutes* and *Bacteroides* were revealed to be the two dominant phyla inhabited in the guts of the three breeds of pigs, yet accounting to varied proportions; *Firmicutes* in Tibetan pigs accounted for 74%, and *Firmicutes* in Barkshire and Landrace pigs accounted for 81 and 91.5%, respectively (Figure 2C), while *Bacteroides* was present as the highest in Tibetan pigs at 17.9%, and the lowest in Landrace pigs, approximately 4.5% (Figure 2D). Moreover, the proportions of *Fibrobacterota*, *Euryarchaeota*, and *Spirochaetota* in the gut bacteria were pronouncedly higher in Tibetan pigs than the other two breeds (Figures 2E,G,I) (*p* < 0.01). However, *Verrucobacteria* and *Desulfobacterota* were higher in Berkshire pigs (Figures 2F,H) (*p* < 0.05 and *p* < 0.01), and *Cyanobacteria* and *Actinobacteriota* in Landrace pigs were higher than the other two breeds (Figures 2J,K) (*p* < 0.05).

**FIGURE 3:**
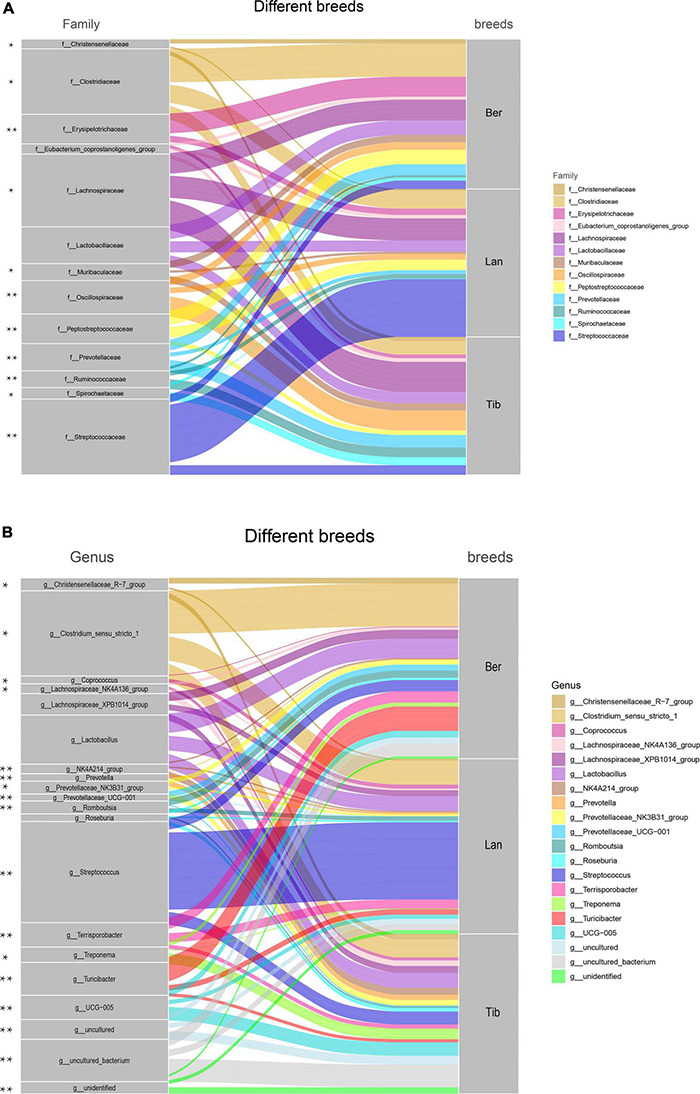
Species composition in the fecal samples of Berkshire, Landrace, and Tibetan pigs. Top 13 dominant family **(A)** and top 20 dominant genus **(B)** were displayed. The color ribbons indicated the associations between families or genus and breeds, of which the colors represented as families or genus. The width of the ribbon reflected the scale of individual connections (the wider the ribbon, the stronger the association) it represented between the families or genus and the breeds. The statistical *p*-values were calculated by Kruskal–Wallis tests. *p < 0.05, **p < 0.01.

Host–gut bacteria association analyses were performed in the top 13 families and 20 genera of the three pig breeds (Figure 3). At the family level, as shown in Figure 3A, pronouncedly higher abundance of family *Streptococcus* was presented in the gut of Landrace pigs than in those of Tibetan and Berkshire pigs, while families *Clostridiaceae*, *Erysipelotrichaceae*, and *Peptostreptococcaceae* were higher in Berkshire pigs (Figure 3A). Greater proportions of *Christensenellaceae*, *Muribaculaceae*, *Prevotellaceae*, *Spirochaetaceae*, *Lachnospiraceae*, *Oscillospiraceae*, and *Ruminococcaceae* resided in Tibetan pigs than in Berkshire and Landrace pigs. Families *Christensenellaceae*, *Muribaculaceae*, *Prevotellaceae*, and *Spirochaetaceae* in Berkshire pigs were higher in abundance than in Landrace pigs. At the genus level, similar to family distribution, *Streptococcus* and *Clostridium* were significantly higher in proportion in the gut flora of Landrace and Berkshire pigs, respectively. In addition, *Romboutsia, Terrisporobacter*, and *Turicibacter* were most abundant in Berkshire pigs. The scale of *Coprococcus* in Berkshire pigs was lower than in the other two groups. *Lachnospiraceae_XPB1014_group* and *Roseburia* in Tibetan pigs and Berkshire pigs were greater in abundance than in Landrace pigs (Figure 3B). The populations of genus *Treponema*, *NK4A214_group*, and *UCG-005* in Tibetan pigs were shown to be significantly higher than those in the other two breeds. The uncultured and unidentified genus presented in Tibetan pigs were obviously higher than in the others (Figure 3B).

**FIGURE 4:**
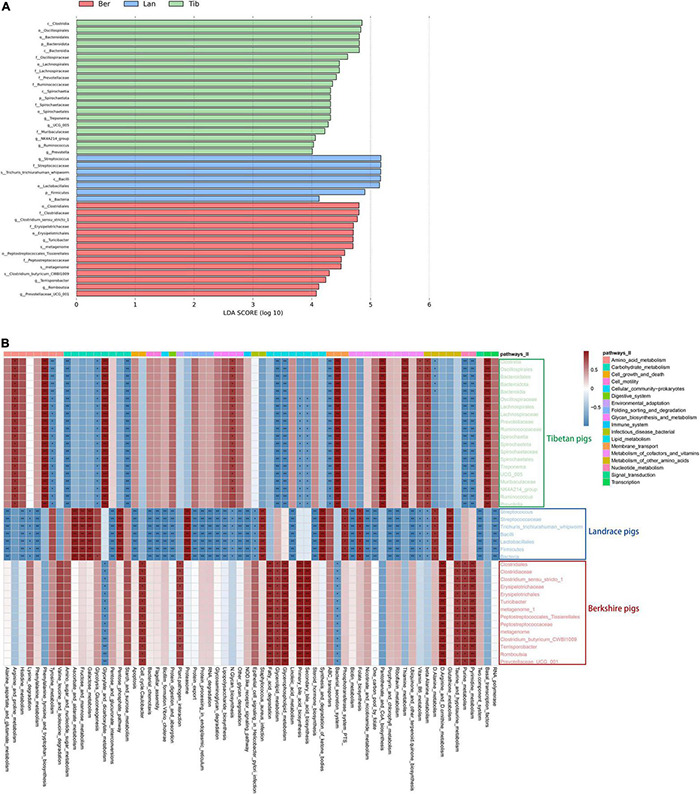
Differences of bacterial species in fecal samples of the three breeds of pigs and heatmap showing the correlation between bacterial species and metabolic pathways. Differences of bacterial species in fecal samples **(A)**. Green, blue, and red represent the bacterial species in Tibetan, Landrace, and Berkshire pigs, respectively. The picture depicts the third-level metabolic pathways annotated against Kyoto Encyclopedia of Genes and Genomes (KEGG). Different colors in the upper part of the picture represent the third-level metabolic pathways annotated to KEGG. Heatmap of correlation between species and metabolic pathways **(B)**. Red represents a positive correlation between species and metabolic pathways, and blue represents a negative correlation. The darker the color, the greater the correlations, *p < 0.05, **p < 0.01.

To further understand the bacterial compositions of gut in detail in each breed of pigs, LDA score was performed using log10 > 4 as the cutoff (Figure 4A); cladogram of the LEfSe analyses is shown in Supplementary Figure 2. As LEfSe analyses, the dominant inhabitants in the gut bacterial communities of the three breeds of pigs were clearly different between each other, which corresponded with the above host–gut bacteria association analysis.

Subsequently, bacterial metabolic functional predictions were conducted (Figure 4B). The seven dominant colonized bacterial species in the gut of Landrace pigs were shown to be highly correlated with the *Staphylococcus_aureus*_infection pathway, and was not correspondent with functions such as Porphyrin_and_chlorophyll_metabolism, Biotin_ metabolism, Nicotinate_and_nicotinamide_metabolism, and Protein_digestion_and_absorption. In contrast, the bacterial species that inhabited the Tibetan pigs’ gut exerted both anti-*Staphylococcus* and anti-*Helicobacter* functions, and mildly correlated with the NOD-like_receptor_signaling_pathway, which is involved in the recognition of dangerous signals and subsequent regulation of the immune responses in the body, which, however, was missing in the gut community of Landrace pigs. Furthermore, the bacterial communities of Tibetan pigs was highly correspondent to the functions of Pantothenate_and_CoA_biosynthesis, Thiamine_metabolism, and Vitamin_B6_metabolism; in particular, species *Clostridia* and *Oscillospirales* showed significantly higher correlations with Vitamin_B6_metabolism (Figure 4B). Besides, the lipid metabolic functions, including Fatty_acid_degradation, Glycerolipid_metabolism, and bile_acid_biosynthesis, were significantly more correlated with the bacterial communities in Berkshire pigs than the other two breeds as shown in Figure 4B.

### Composition and Functional Potential of Gut Bacteria in Tibetan Pigs at Different Growth Stages

**FIGURE 5:**
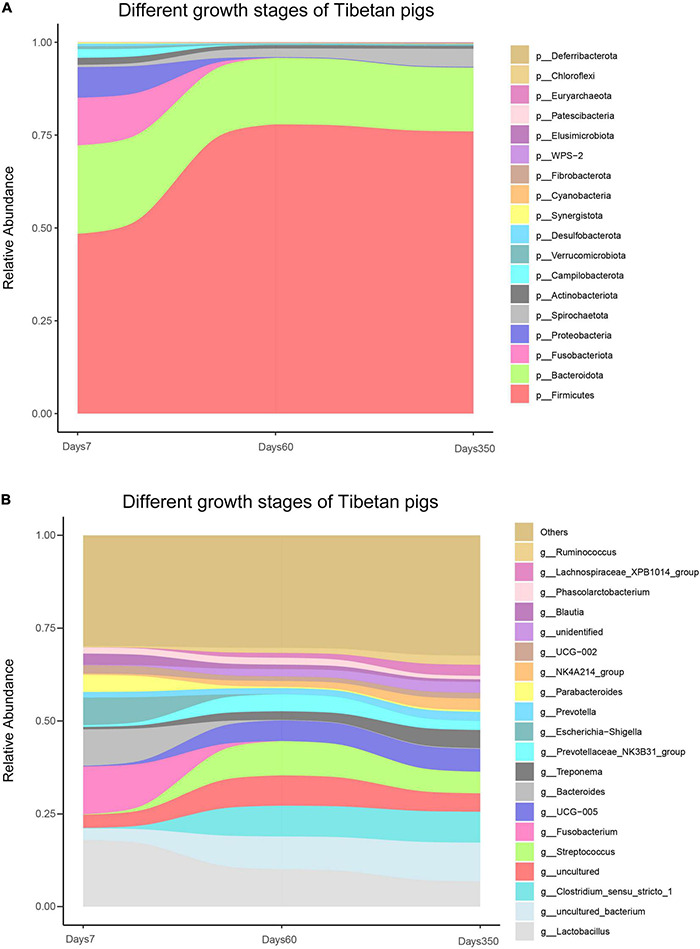
Bacterial composition of Tibetan pigs at different growth stages. Stream-graph displaying the relative abundance of bacterial taxa in days 7, 60, and 350 of Tibetan pigs. Species composition at phylum level **(A)** and top 20 genus **(B)**. Low abundant taxa were grouped as “others”.

To understand the dynamics of bacterial communities in Tibetan pigs, the gut bacteria were determined at days 7, 60, and 350, respectively. Our results showed that 1,640 OTUs were identified that resided through the three time points in total. Generally, the number of OTUs increased with the growth of the host: 542, 1,206, and 1,390 OTUs were determined at the above time points separately (Supplementary Figure 3). Moreover, the scale of individual population of bacterial species was experiencing dynamic variations in the process of the growth of pigs as observed in Figure 5A. At the phylum level, *Firmicutes* exhibited a distinct pattern throughout the experimental period, displaying a rising trend in the beginning till around day 60, then started to decline gently, accounting to 48.4, 77.9, and 76.0% at days 7, 60, and 350, respectively. The proportions of *Bacteroidota*, *Proteobacteria*, *Actinobacterota*, *Fusobacteriota*, *Campilobacterota*, *Desulfobacterota*, and *Verrucomicrobiota* had a higher initial relative abundance during the first 7 days, then decreased with the growth of the host, and *Patescibacteria* decreased, while the specific densities of *Spirochaetota*, *Fibrobacterota*, *WPS-2*, *Elusimicrobiota*, and *Deferribacterota* showed increases in the pigs as time passes. Moreover, *Cyanobacteria* and *Chloroflexi* possessed the highest levels of colonized population within the initial 60-day age of pigs.

At the genus level, the analysis showed that the colonized populations of *Clostridium_sensu_stricto_1*, *UCG-005*, *Treponema*, *Prevotella*, *NK4A214_group*, *Lachnospiraceae_XPB1014_group*, *Ruminococcus*, unidentified, and other rare species in pigs increased, while those of *Lactobacillus*, *Fusobacterium*, and *Prevoichilauace-ae_NK3* reduced with the aging of the pigs (Figure 5B). Moreover, Uncultured_bacterium and *Streptococcus* showed a fluctuation of initial rising and then a decreasing trend. *Bacteroides*, however, showed a reverse trend (Figure 5B).

**FIGURE 6:**
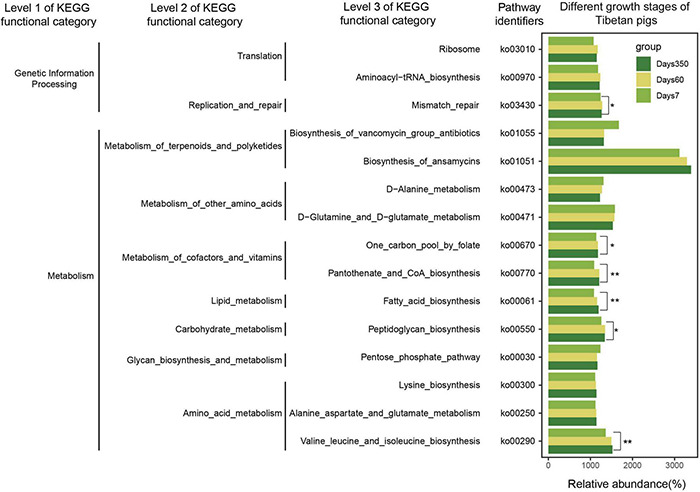
Bacterial functional prediction of Tibetan pigs at three growth stages. The picture shows three levels of KEGG functional category. The gradient colors represent three growth stages. Significant differences were calculated by Kruskal–Wallis tests, *p < 0.05, **p < 0.01.

Furthermore, bacterial functional predictions using PICRUSt were performed. Three layers of KEGG functional categories and the top 15 pathways were taken into account, covering the information of genetic processing and metabolisms of lipids, amino acids, and carbohydrates. As shown in Figure 6, significantly high abundance of bacterial flora was correlative with the biosynthesis of ansamycin throughout the growth of the pigs. Pronounced increases of bacterial species were related to Valine_leucine_and_isoleucine and Fatty_acid_biosynthetic pathways as the pigs grew. In addition, it was shown that the abundance of gut flora correlated with the biosynthesis of vancomycin and was significantly higher at day 7 than those of days 60 and 350. The bacterial abundances correlated to the Pentose phosphate pathway and One carbon pool by folate pathways were initially exhibited as decreasing trends. Then, with the aging of the pigs, more bacteria became involved, whereas the tendency reversed when it came to the Pantothenate and CoA biosynthesis and Ribosome procession.

**FIGURE 7:**
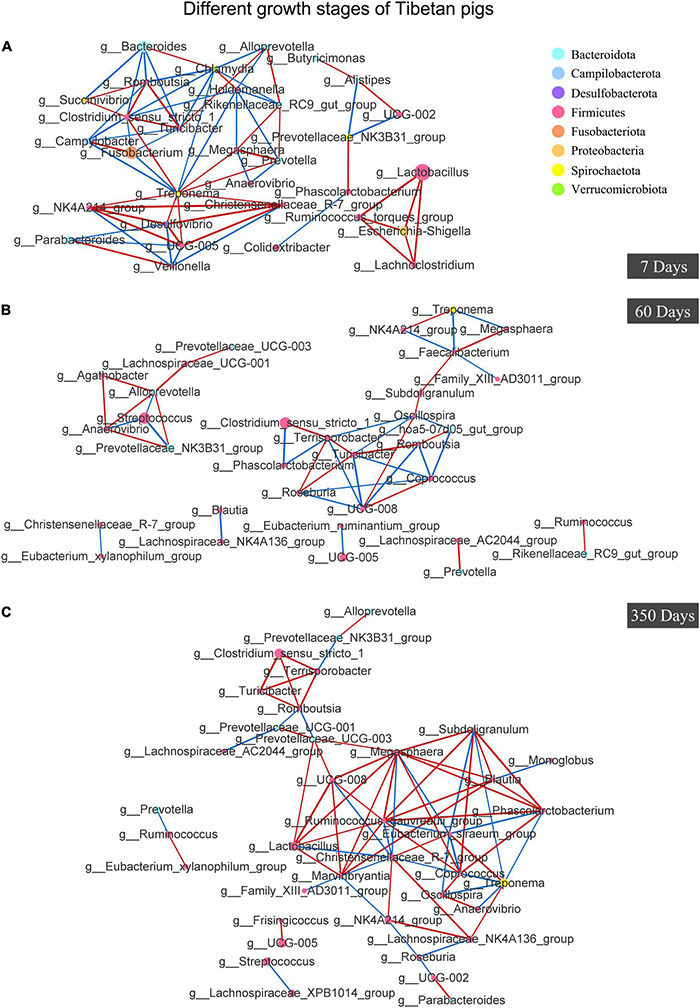
Analysis of Co-occurrence Network of gut bacteria in Tibetan pigs at different growth stages. Co-occurrence Network of Tibetan pigs at day 7 **(A)**, day 60 **(B)**, and day 350 **(C)**. Individual color of dots represented the phyla in the gut. The size of nodes was proportional to the relative abundance of genus. Red lines stood for positive correlations between species, and blue lines represented negative correlations between species. The thickness of the lines indicated the size of the correlation coefficient value.

As for the colony interactions shown in Figure 7, the network of gut bacteria of suckling pigs at day 7 was clearly distributed into three clusters, one of which, with g_*Escherichia-Shigella* as the core genera, extended to comprise g_*Lactobacillus*, g_*Lachnoclostridium*, and g_*Ruminococcus*_*torques*_group in it (Figure 7A). However, the symbiosis network of 60-day pigs was shown to be more independent and simpler (less connected nodes and edges), and not as complicatedly weaved as observed in the pigs at day 350 (more connected nodes and edges) (Figure 7B). On day 350, the colonized gut flora was shown a high taxonomic connection in the Tibetan pigs, except that a few genera were separately connected forward or backward (Figure 7C).

### Composition and Functional Potential of Gut Bacteria Between Two Genders of Tibetan Pigs

The feces of boars and sows in pregnant and lactate phases were collected; 710 shared OTUs were identified among these boars and sows, with 1,016, 984, and 910 OTUs for boars, pregnant sows, and lactating sows, respectively (Supplementary Figure 4).

**FIGURE 8:**
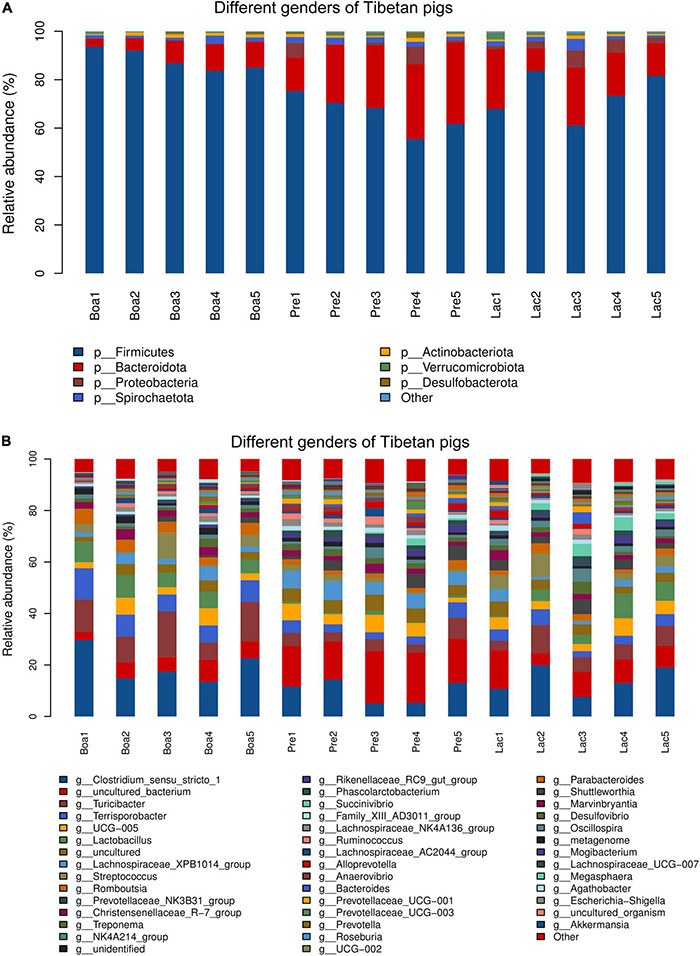
Relative abundance of bacterial species residing in the guts of boars, pregnant sows, and lactating sows. At the phylum level **(A)** and at the genus level **(B)**.

At the phylum level, *Firmicutes* was the dominant bacteria in both genders. Specifically, a higher abundance of *Firmicutes* and lower *Bacteroidota*, *Proteobacteria*, and *Spirochaetota* inhabited in boars compared to those in sows. The abundances of *Bacteroidota* and *Spirochaetota* in pregnant sows were higher than those of lactating sows. However, the bacterial phylum *Verrucomicrobiota* tended to colonize the gut of lactating rather than pregnant sows, and the least in the boars. *Actinobacteriota*, however, exhibited a reverse trend (Figure 8A).

At the genus level, the abundances of colonial *Clostridium_sensu_stricto_1*, *Turicibacter*, *Terrisporobacter*, *Lactobacillus*, and *Romboutsia* in boars were significantly higher than those in sows’ gut flora. The genus *Succinivibrio* presented the most in lactating sows and least in boars. Uncultured_bacterium exhibited as the most in pregnant sows and least in boars (Figure 8B).

**FIGURE 9:**
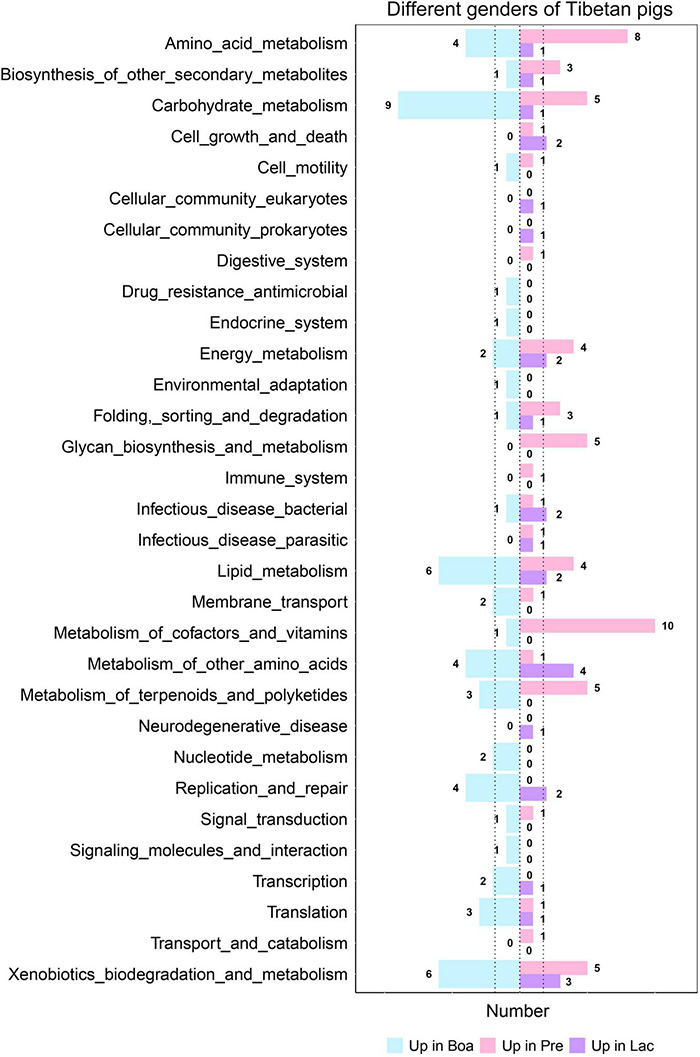
Correlations of gut bacteria in boars, and pregnant and lactating sows to each level 3 KEGG pathway. The figure shows the number of pathways with the most significant correlation among the three groups.

Bacterial functional predictions against 31 relevant KEGG pathways were performed to further understand the effects of gender on gut flora colonization (Figure 9). Gut bacteria of boars were more correlative to the pathways of metabolisms of carbohydrate, lipid, drug resistance, environmental adaptation, and xenobiotics biodegradations; pregnant sows were the next for these metabolisms, except for the metabolisms of drug resistance and environmental adaptation (Figure 9). However, the bacterial communities of pregnant sows were most correlated with the pathways of metabolisms of amino acid, digestion, energy, glycan biosynthesis and metabolism, immune responses, and vitamins and its cofactors. In particular, the correlation index reached 10 for the metabolism of cofactors and vitamins, significantly higher than those for boars and lactating sows. The gut bacteria in lactating sows were more related to the infectious_disease_bacterial pathway than others. In particular, the relations were significantly lower in the pathways including amino acid, carbohydrate, and lipid metabolism in lactating sows (Figure 9). In addition, the intestinal flora of boars was the most respondent to the biosynthesis of ansamycins (*p* < 0.05) (Supplementary Figure 5).

## Discussion

Compared to previous research (Wang et al., 2012; Jiang et al., 2018; Zeng et al., 2020), the Tibetan pigs under semi-grazing were selected as research subjects. The pigs are introduced and raised in the other areas and circulated in the market. Moreover, they live in semi-grazing conditions and are not fed commercial feed. Perhaps in China, such a “second-generation Tibetan pig” is more likely to be the future development trend. In this study, we determined the bacterial composition of the gut microbiota in Tibetan pigs, and those of Berkshire and Landrace pigs were used for comparison. Furthermore, we found that growth stage, gender, pregnancy, or lactation of sows all affect gut bacteria of Tibetan pigs.

As expected, the structure of gut community in the Tibetan pigs was more complex than the other two breeds. Previous studies had demonstrated that the gut microbiota played an important role on the adaptability of yak to harsh environments of the Qinghai-Tibet Plateau (Guo et al., 2021). Tibetan pigs, similarly, is a breed of highland pig. Studies have supported the hypothesis that the given high-altitude environment helps to shape particular microbiota colonized in the gut, in turn facilitating Tibetan pigs to adapt to the harsh environment (Zeng et al., 2020). The relationship between intestinal bacteria and environmental adaptability of Tibetan pigs in the introduction area remains to be explored.

The Berkshire pig was known for its high-quality meat (Jang et al., 2018) and adaptation to semi-grazing conditions. Landrace pigs, on the contrary, claimed to have a high growth rate (Tang et al., 2007). Comparatively, the gut bacterial structure of the Tibetan pigs was supposed to be more similar to that of Berkshire pigs. Our data showed that the abundance of phylum *Firmicutes* colonized in the gut of Tibetan pigs was significantly lower, whereas *Bacteroides* was much higher than in the other two breeds. The roles of intestinal *Firmicutes* and *Bacteroides* on host’s obesity were initially established in 2005, an observation based on the investigation of the microbiota of genetically modified, lean, and wild-type mice, which demonstrated that the colonized *Bacteroidetes* was 50% lower in obese animals than their counterpart in lean mice (Tang et al., 2007). In addition, it was found that a low *Firmicutes*/*Bacteroidetes* ratio was associated with the lean phenotype and a healthy cardiovascular and balanced immune system (Nicholson et al., 2012; Tang et al., 2017). Phylum *Fibrobacteriota* comprises many rumen bacteria, allowing for the degradation of cellulose (Ransom-Jones et al., 2012, 2014). In this study, a higher proportion of *Fibrobacterota* was identified in the gut of Tibetan pigs, which might be ascribed to its herbivory. In addition, human clinical data showed that the abundance of *Verrucomicrobia* in human intestine was inversely associated with the state of host bacterial infections (Dubourg et al., 2013). This may indirectly reflect the high disease-resistant capacity of Tibetan pigs, in which *Verrucomicrobia* had been a long inhabitant. Our data also showed that a number of bacterial families that correlated with anti-inflammation, nutrient metabolism, and absorption pathways resided in the gut of Tibetan pigs in high abundance, such as the probiotic *Christensenellaceae* and *Muribaculaceae*, which had been confirmed to be negatively related to inflammatory and metabolic diseases (Tavella et al., 2021); *Lachnospiraceae* and *Prevotellaceae*, which were related to the synthesis of short-chain fatty acids (SCFAs) responsible for the protection of intestinal mucosae (Purushe et al., 2010; Laffin et al., 2019); and *Methanobrevibacter*, which was an important player in the hydrogen nutrient methane production pathway (St-Pierre and Wright, 2013); moreover, *Muribaculaceae* (S24-7) was shown to be capable of inhibiting inflammatory responses in the animal’s intestinal tract, playing an important role in maintaining intestinal health (Ormerod et al., 2016). *Ruminococcaceae*, a producer of butyric acid providing energy for intestinal epithelial cells, is involved in the maintenance of the health of intestine (Serpa et al., 2010) and exerted negative effects on colorectal cancer (Dayama et al., 2020). Besides, *Treponema bryantii* of genus *Treponema*, the most abundant in phylum *Spirochetes*, was identified to be colonized in high abundance in Tibetan pigs and possessed the ability to inhibit intestinal pathogenic bacteria, such as *Escherichia coli* and *Salmonella* (Edrington et al., 2012). All these data evidenced the high capability of disease prevention of Tibetan pigs.

Furthermore, bacterial functional analysis also supported that the gut bacteria of Tibetan pigs was more robustly correspondent to the anti-infection metabolism or pathways. The microflora residing in Landrace pigs was significantly correlated with *Staphylococcus aureus* infections, and the risk of streptococcal infection was high. In contrast, the bacterial communities of Tibetan pigs was vigorously correlated with vitamin B1, B5, and B6 metabolism and synthesis, which facilitated to strengthen its immune system, especially anti-inflammatory responses (Du et al., 2020). The NOD-like receptor was a key factor involved in the regulation of the host’s native immune responses and was identified to be significantly correlated with the bacteria in Tibetan pigs. Long-term consumption of green roughage may contribute to the shape of disease-resistant characterization of the gut bacteria in Tibetan pigs.

Taxonomic richness displayed a positively linear correlation with the growth of pigs, which was consistent with previous studies (Jurburg and Bossers, 2021). Specifically, with the increase of age, the intestinal bacteria of Tibetan pigs became more and more abundant. In addition, previous studies revealed that increases in the prevalence of *Fusobacteria* were related to colorectal cancer (Kelly et al., 2018), malnutrition (Pham et al., 2019), impaired human immune recovery (Lee et al., 2018), and biological disorders in suckling pigs (Huang et al., 2019). Our study showed that the abundance of *Fusobacteriota* gradually declined with the aging of the hosts until they disappeared from the gut bacteria on day 360 of Tibetan pigs.

As expected, gut bacteria of Tibetan pigs were more correlated with the perfection of metabolic functions with age; metabolisms of terpenoids and polyketides, amino acids, and lipids, especially the ability of biosynthesis of ansamycins of the host, were enhanced, which could strengthen the anti-microbial and anti-cancer ability. In addition, the genetic processing was maximized at 60 days of age of the pigs.

Extensive studies showed that diet affected the host’s gut microbiota; a stable diet can stabilize the gut microbiota to a certain extent (Han et al., 2020; Gao et al., 2021). Comparatively, the taxonomic relations of gut microbiota in 60-day pigs was unstable, which may be caused by weaning. Previous reports also found high variability in PD whole tree and Shannon index in 56-day-old Tibetan minipigs (Jiang et al., 2018). Instead, a stable gut bacteria relationship was exhibited in 360-day pigs, which were supposed to have more stable body functions and immune responses comparatively (Figure 7).

Gender exerted effects on the abundances of the bacterial families residing in the gut of Tibetan pigs. Our analysis showed that the dominant bacteria phyla were the same; the population sizes, however, appeared to be different across boars’, pregnant sows’, and lactating sows’ gut bacteria. Of these, *Lactobacillus* was shown to be significantly higher in boars than sows. *Lactobacillus* species are probiotics, and considered to be beneficial to hosts’ health (Gareau et al., 2010). The high abundance of *Succinivibrio* in boars’ gut bacteria was also a signature of its high adaptability. *Succinivibrio* species was characterized as high capacity of degradation of cellulose and positively correlated with IL-10, a crucial immunomodulatory cytokine. In contrast, decreases in the abundance of *Succinivibrio* may help maintain the mucosal barrier and prevent chronic immune activation in the natural host (Jasinska et al., 2020) during Simian immunodeficiency virus infection. The exact function of this bacterium needs further study.

Vitamins in the diet were supposed to have beneficial effects on women’s fertility (Skoracka et al., 2021). Interestingly, the gut bacteria in pregnant sows appeared to be more correlated to the metabolism of cofactors and vitamins than the other two groups, and lactating sows appeared to have higher responses to bacterial infections, which may be more beneficial to improve the levels of maternal antibodies.

In conclusion, the bacterial composition of Tibetan pigs exhibited a distinct structure, many of which were uncultivated and unidentified bacteria families. These may represent new prokaryotic taxa and increase the phylogenetic diversity of the microbial tree of life (Zhou et al., 2020). In addition, despite individual growth stages, genders and physiological periods difference, bacterial populations of Tibetan pigs exhibited a dynamic equilibrium with varied abundance. This pilot study on the gut bacterial communities in Tibetan pigs may shed light on how we could improve our human adaptability *via* modulating microecology.

## Data Availability Statement

Raw sequencing data obtained were deposited in the database SRA under accession number PRJNA768095.

## Ethics Statement

All experiments and procedures were approved by Jilin Agricultural University. All animal experiments in the study complied with the regulations of the Animal Protection and Ethics Committee of Jilin Agricultural University (JLAU20210111001). Written informed consent was obtained from the owners for the participation of their animals in this study.

## Author Contributions

W-TY, C-FW, G-LY, and C-WS designed the research. HN, X-ZF, DZ, and H-LC performed the research. H-BH, Y-LJ, NW, J-ZW, XC, and YZ analyzed the data. W-TY, C-FW, and G-LY supported the research. HN and W-TY wrote the manuscript. All authors contributed to the article and approved the submitted version.

## Conflict of Interest

The authors declare that the research was conducted in the absence of any commercial or financial relationships that could be construed as a potential conflict of interest.

## Publisher’s Note

All claims expressed in this article are solely those of the authors and do not necessarily represent those of their affiliated organizations, or those of the publisher, the editors and the reviewers. Any product that may be evaluated in this article, or claim that may be made by its manufacturer, is not guaranteed or endorsed by the publisher.
