# Supplementary material for: Gut Bacterial Composition and Functional Potential of Tibetan Pigs Under Semi-Grazing
Source: Front Microbiol. 2022 Apr 7;13:850687. doi: 10.3389/fmicb.2022.850687 (PMC9023118; doi:10.3389/fmicb.2022.850687)
Supplement: Supplementary file 1 [file Data_Sheet_1.docx]

Supplementary Material


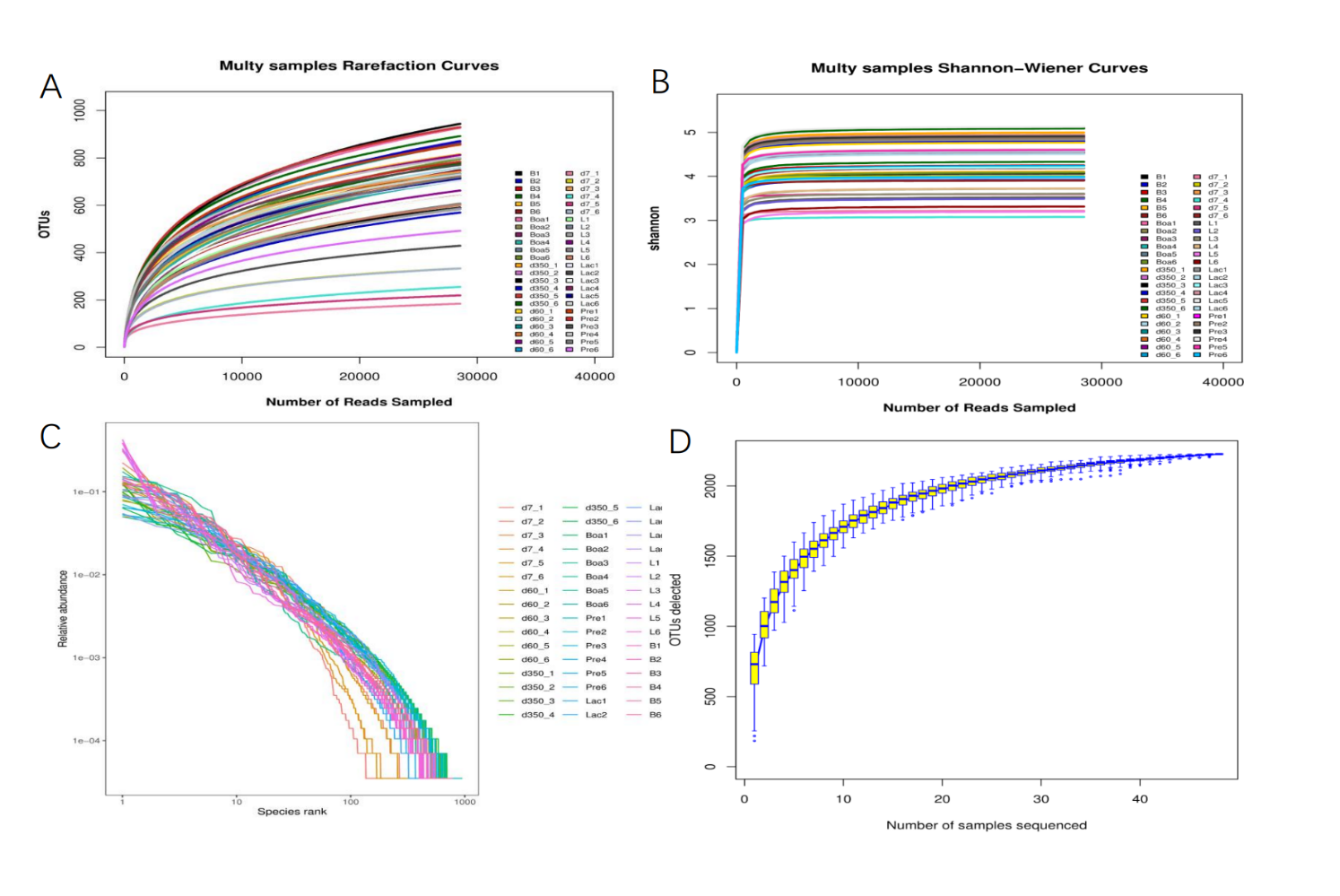


**Supplementary Figure 1. Curves for the OTUs obtained from 48 samples.** Good's coverage analysis of sequencing data. The Rarefaction curve (A), Shannon-Wiener curve (B), and Rank-Abundance curve (C)，species accumulation curve (D). B, samples from the Berkshire pigs; L, samples from the Landrace pigs; d7, samples from the 7 days old Tibetan pigs; d60, samples from the 60 days old Tibetan pigs; d350, samples from the 350 days old Tibetan pigs; Boa, samples from the boars of Tibetan pigs; Pre, samples from the pregnant sows of Tibetan pigs; Lac, samples from the lactating sows of Tibetan pigs.

**
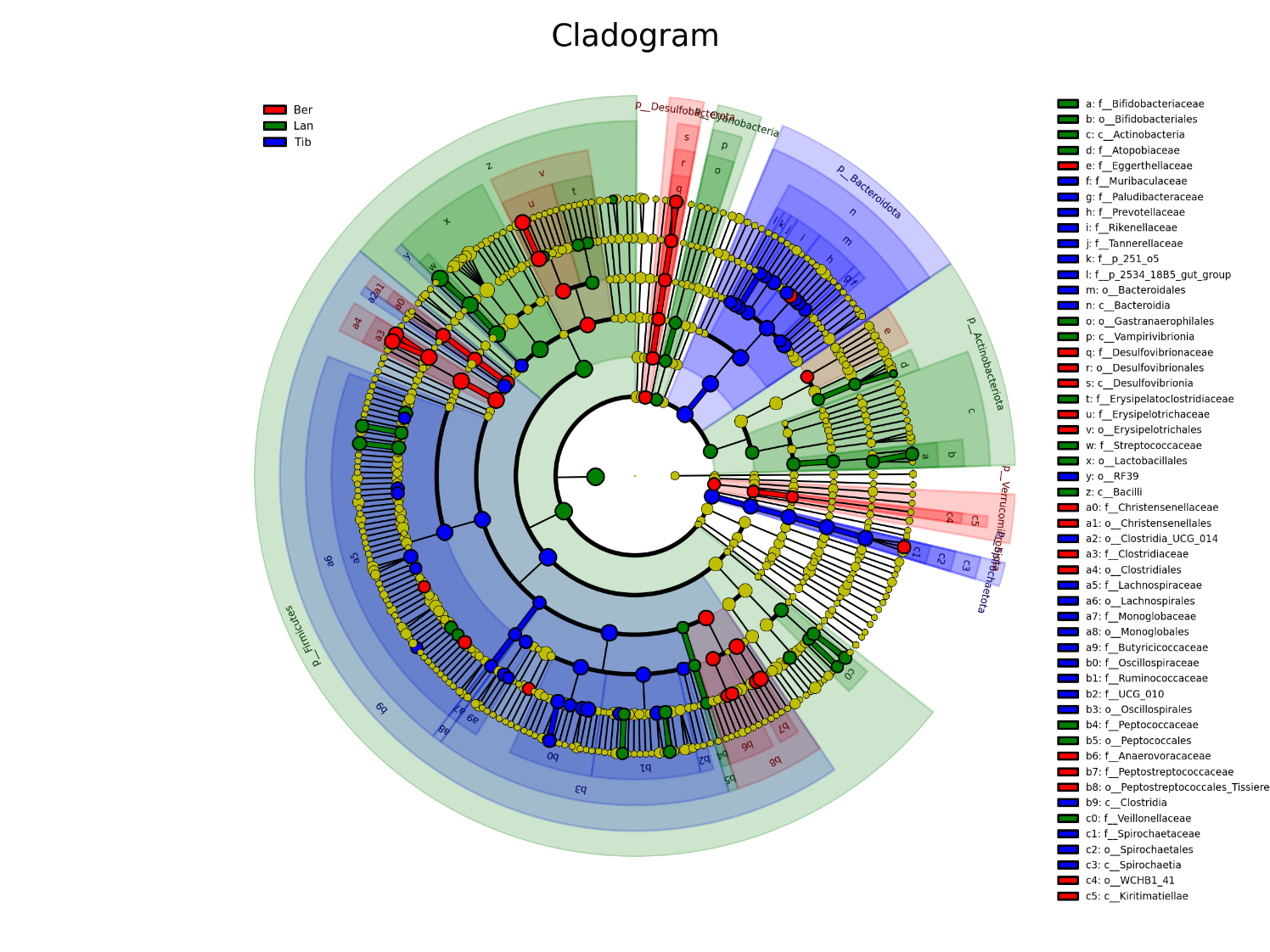
** **Supplementary Figure 2. Cladogram of the LEfSe analyses of the gut bacteria in different**

**breeds.**


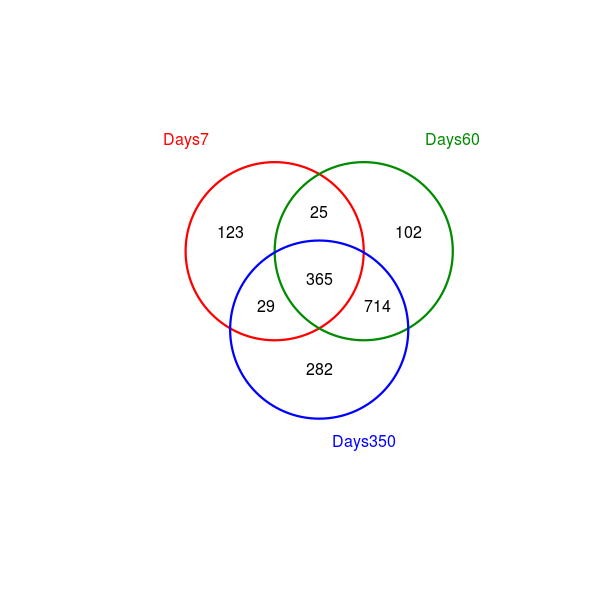
**Supplementary Figure 3. Venn map of Tibetan pigs at different growth stages.**


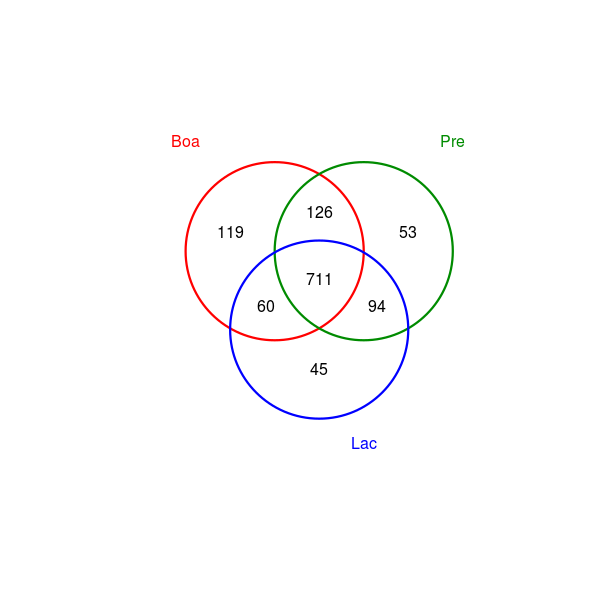
**Supplementary Figure 4. Venn map of Tibetan pigs at different genders.**


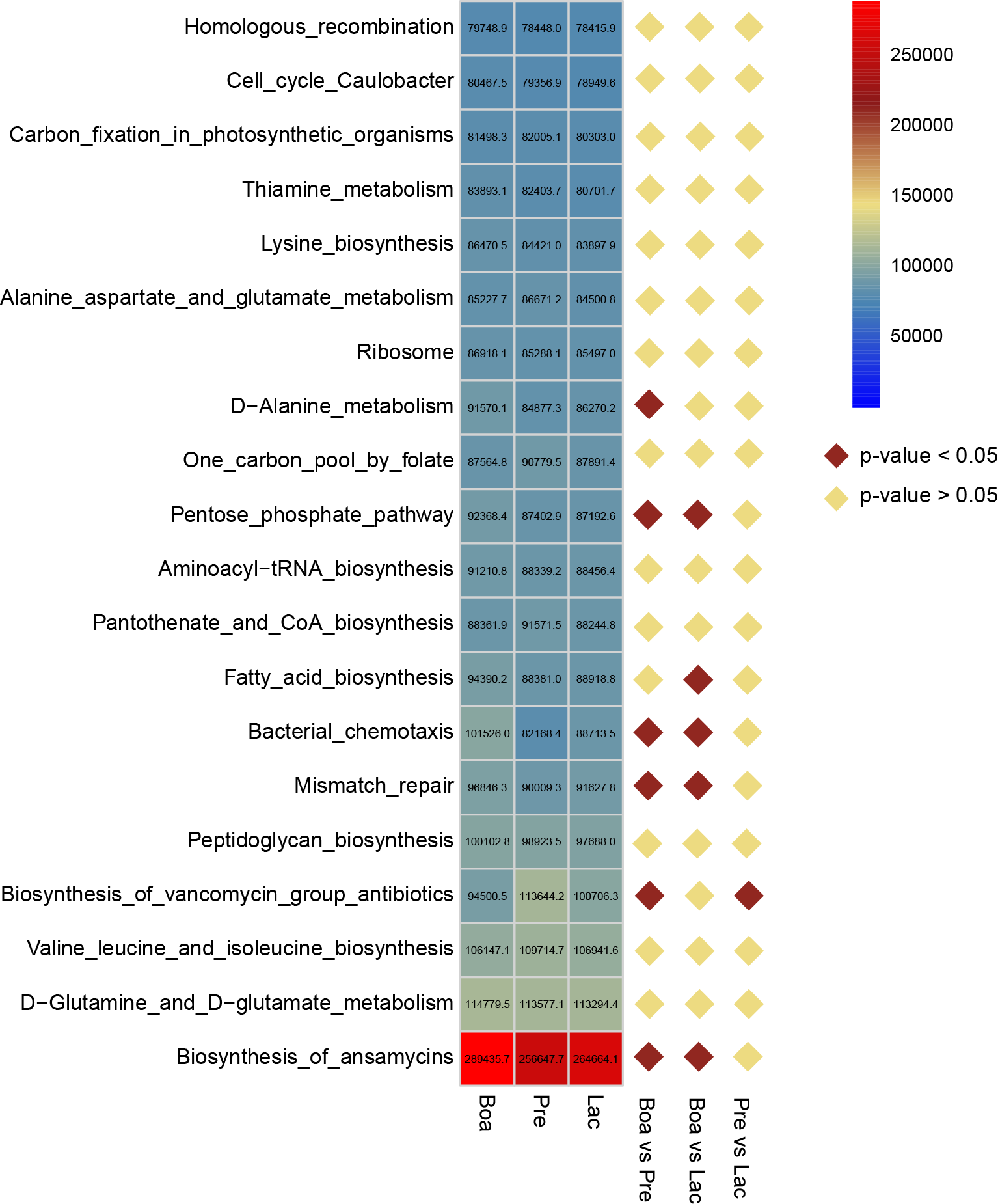


**Supplementary Figure 5. Heatmap of top 20 Level 3 of KEGG functional category.** *P*-values were obtained using a t-test. Boa, boars of Tibetan pigs; Pre, pregnant sows of Tibetan pigs; Lac, lactating sows of Tibetan pigs.
